# Targeted interventions of the Avahan program and their association with intermediate outcomes among female sex workers in Maharashtra, India

**DOI:** 10.1186/1471-2458-11-S6-S2

**Published:** 2011-12-29

**Authors:** Mandar M Mainkar, Dilip B Pardeshi, Jayesh Dale, Sucheta Deshpande, Shirin Khazi, Abhishek Gautam, Prabuddhagopal Goswami, Rajatashuvra Adhikary, Shreena Ramanathan, Bitra George, Ramesh S Paranjape

**Affiliations:** 1 National AIDS Research Institute (ICMR), Pune, India; 2 FHI India, New Delhi, India

## Abstract

**Background:**

Avahan, the India AIDS Initiative has been a partner supporting targeted interventions of high risk populations under India’s National AIDS Control Organisation (NACO) since 2004 in the state of Maharashtra. This paper presents an assessment of the Avahan program among female sex workers (FSWs) in Maharashtra, its coverage, outcomes achieved and their association with Avahan program.

**Methods:**

An analytical framework based on the Avahan evaluation design was used, addressing assessment questions on program implementation, intermediate outcomes and association of outcomes with Avahan. Data from routine program monitoring, two rounds of cross-sectional Integrated Behavioural and Biological Assessments (IBBAs) conducted in 2006 (Round 1- R1) and 2009 (Round 2 – R2) and quality assessments of program clinics were used. Bi-variate and multivariate analysis were conducted using the complex samples module in SPSS 15® (IBM, Somers NY).

**Results:**

The Avahan program achieved coverage of over 66% of FSWs within four years of implementation. The IBBA data showed increased contact by peers in R2 compared to R1 (AOR:2.34; p=0.001). Reported condom use with clients increased in R2 and number of FSWs reporting zero unprotected sex acts increased from 76.2% (R1) to 94.6% (R2) [AOR: 5.1, p=0.001].

Significant declines were observed in prevalence of syphilis (RPR) (15.8% to 10.8%; AOR:0.54; p=0.001), chlamydia (8% to 6.2%; AOR:.0.65; p=0.010) and gonorrohoea (7.4% to 3.9; AOR:.0.60; p=0.026) between R1 and R2. HIV prevalence increased (25.8% to 27.5%; AOR:1.29; p=0.04). District-wise analysis showed decline in three districts and increase in Mumbai and Thane districts.

FSWs exposed to Avahan had higher consistent condom use with occasional (94.3% vs. 90.6%; AOR: 1.55; p=0.04) and regular clients (92.5% vs. 86.0%; AOR: 1.95, p=0.001) compared to FSWs unexposed to Avahan. Decline in high titre syphilis was associated with Avahan exposure.

**Conclusion:**

The Avahan program was scaled up and achieved high coverage of FSWs in Maharashtra amidst multiple intervention players. Avahan coverage of FSWs was associated with improved safe sexual practices and declines in STIs. Prevalence of HIV increased requiring more detailed understanding of the data and, if confirmed, new approaches for HIV control.

## Background

India is the seventh largest country geographically and the second most populated country in the world. The HIV epidemic emerged in India almost a decade later than in the rest of the world. HIV rates in India increased quite rapidly in the 1990’s. The government of India estimated that about 2.31 million people are living with HIV (1.8-2.9 million) with adult prevalence of 0.34 percent [1]. India is highly heterogeneous and the epidemic is largely concentrated among most at risk populations in six states: Andhra Pradesh, Karnataka, Maharashtra, Tamil Nadu in the south and Manipur and Nagaland in the north east [1,2].

Maharashtra, the second most populated Indian state and home to Mumbai, India’s economic capital, is located on the west coast of India. It was one of the earliest states to be affected by HIV [2-4]. The first cases of HIV were detected in Mumbai in 1986 and, by 2003, about 21% of all reported AIDS cases in India were from Maharashtra [5]. The epidemic in Maharashtra is similar to other epidemics in Asia, having begun among female sex workers (FSWs), men who have sex with men (MSM) and clients of sex workers [6,7].

The Indian National AIDS Control Organisation (NACO) has been coordinating and implementing India's national response for HIV control since 1992 [8]. One component of the HIV response has been sustained interventions for FSWs. Avahan, the India AIDS Initiative, with the objective of halting and reversing the spread of HIV, has been a major partner in supporting NACO prevention interventions since 2004 [9]. Based on the epidemiology of HIV in India in 2003 and available program coverage data, Avahan decided to work in the six high prevalence states at that time including Maharashtra [9]. Avahan’s program strategies were designed to achieve high coverage (target of 80%) in selected sites through delivery of a package of proven prevention services to FSWs, addressing proximal and distal determinants of HIV risk [10]. The key program elements were similar to that of NACO and included peer-based outreach education, clinical services for managing STIs, promotion and distribution of condoms, community mobilization and building an enabling environment [9].

**Figure 1:**
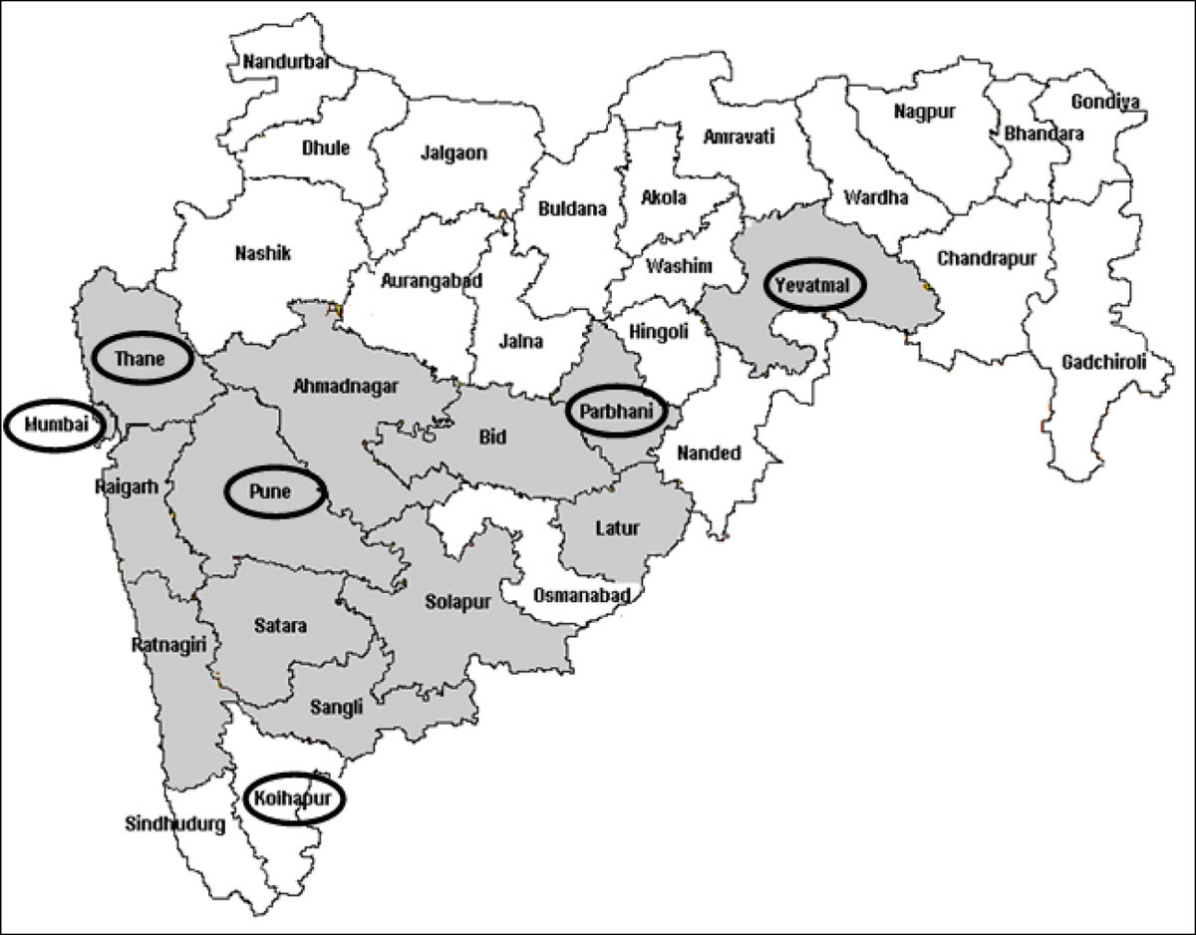
Maharashtra District Map with Avahan Intervention and IBBA districts

In Maharashtra, the Avahan program focused on strengthening the existing program for FSWs and MSM. In addition, Avahan directly supported program linked STI clinics in contrast to most other services that used referral to government clinics to provide services. Since the early 1990s, HIV prevention and control programs have been implemented in most districts by the Maharashtra State AIDS Control Society (MSACS) and, in the district of Mumbai, by the Mumbai District AIDS Control Society (MDACS) [11]. Sixteen districts out of thirty five were chosen for Avahan program implementation in conjunction with these State AIDS Control Societies. Figure 1 gives the geographic coverage of Avahan interventions in the state of Maharashtra. Table 1 provides the details of the Avahan districts and the percentage of Avahan’s planned coverage of the estimated target population of FSWs in the district. In four of the districts, Avahan was the sole source (100% coverage) for interventions, whereas in the other districts, Avahan interventions covered the targeted high-risk populations as a major, equal or minor program (based on the percentage of coverage of estimated FSWs in the district) along with the government and other agencies.

**Table 1:** District-level Avahan intended coverage and history of intervention in Maharashtra

Districts	District population^1^ (thousands)	Estimated size of FSW in districts^2^	Avahan intended coverage	History / type of intervention coverage*	IBBA sample	
					
					R-I	R-II
Ahmednagar	4,088	9,57	100%	First and solo		
Beed	2,161	2,280	87%	Not first but major		
Jalgaon	3,679	8,80	66%	Not first but major		
Kolhapur	3,515	1,109	41%	Not first but equal	115	190
Latur	2,080	1,830	51%	Not first but major		
Mumbai brothel	13,830	23,483^+^	43%	Not first but major	407	395
Mumbai street			42%	Not first but major	394	385
Nandurbar	1,309	4,09	100%	First and solo		
Nashik	4,993	8,19	63%	Not first but major		
Parbhani	1,527	2,135	72%	Not first but major	367	303
Pune brothel	9,924	7,223^+^	100%	Not first but equal	404	403
Pune street			81%	Not first but major	257	266
Sangli	2,583	2,020	100%	Not first but solo		
Satara	2,796	5,06	76%	Not first but solo		
Solapur	3,849	6,050	79%	Not first and only clinical services		
Thane brothel	8,131	12,704^+^	27%	Not first but major	401	384
Thane street			100%	Not first but major	394	395
Yavatmal	2,460	9,69	100%	First and solo	153	157

**Source: 1-**Census 2001; **2-**District mapping data **3-**Avahan CMIS 2009

**First and solo: 1)** Prior to Avahan (i.e. 2003), there was no intervention for FSWs; **2)** Currently, Avahan is the only intervention in the district.

**Not first but major:** Prior to Avahan (i.e. 2003), FSW interventions existed in the districts and currently Avahan covers more than 50% of the mapped FSW population in the district

**Not first but equal:** Prior to Avahan (i.e. 2003), FSW interventions existed in the districts and currently Avahan covers 30-50% of the mapped FSW population in the district

**Not first and only clinical services:** Prior to Avahan (i.e. 2003), FSW interventions existed in the districts and Avahan provides only clinical services in the district

+ Size estimate by typology was not available

The typology of FSWs in Maharashtra is complex and includes a large number of both street-based, brothel- or lodge-based sex work and others [12]. Particularly centres such as Mumbai, Thane and Pune, the large numbers of FSWs of each typology, the scale of commercial sex and having other intervention agencies implementing programs for high risk groups warranted complementary interventions and strategies to reach street-based and brothel-based FSWs to ensure coverage [13]. In all implementation districts in the state, Avahan coverage included all the prevalent typologies of FSWs, street and brothel-based FSWs.

The objective of this paper is to present early results from the assessment of Avahan’s HIV prevention program among FSWs in Maharashtra, using as its basis the Avahan evaluation framework [14] which adopted an approach that was based on the concepts of adequacy and plausibility for public health programme evaluations [15]. The paper examines the scale, intensity and quality of Avahan program coverage, changes in intermediate outcomes, STIs including HIV, and the association of the Avahan program exposure to changes in the intermediate outcomes in Maharashtra.

## Methods

### Analytical framework for assessment

An analytical framework for this assessment draws directly from the Avahan program evaluation design [14,16]. The framework addresses the assessment questions step-by-step following the logical sequence of program implementation (process and output indicators), intermediate outcomes and contributions of Avahan [17]. The specific aims in this analysis were to: (a) examine the scale, intensity (based on availability and utilization of services), and quality of Avahan coverage; (b) assess the intermediate outcome of consistent condom use; (c) assess the changes in prevalence of STIs and new HIV infections among FSW; and d) examine the association of exposure to Avahan programming with changes in condom use and STI and HIV prevalence.

### Data sources

The present analysis used different data sources: Avahan routine program monitoring data [16], two rounds of cross-sectional surveys conducted among FSWs (termed Integrated Behavioural and Biological Assessments – IBBAs) [18,19], condom sales data from social marketing efforts [20], and external quality assessments conducted in Avahan program STI clinics over time [21].

#### a. Avahan routine program monitoring data

Avahan developed a computerized management information system (CMIS) through the course of its program implementation [16,22] which provided data on program inputs, infrastructure, outreach, and clinical service utilization. Data were aggregated at the NGO level and sent to the state level lead implementing partner. Key variables were further aggregated using a consolidated CMIS. Program monitoring data were used to assess trends in the coverage and uptake of program services from January 2005 through March 2009 [16]. This analysis included 16 of the 35 districts in Maharashtra, where Avahan implemented interventions from 2005 to 2009.

#### b. Integrated Behavioural and Biological Assessments

Two rounds of cross sectional IBBAs were undertaken among FSWs in six of the Avahan intervention districts of Maharashtra, namely Kolhapur, Mumbai, Pune, Parbhani, Thane, and Yevatmal [18]. District selection was purposeful based on the size of FSW population and representativeness of different socio-cultural regions of the state. Round I was conducted between March and December 2006 and Round II between March and December 2009 using the same survey methodology. Field work was conducted by research agencies under the guidance and supervision of the implementing Institute, National AIDS Research Institute (NARI) an affiliate of Indian Council of Medical Research (ICMR), after training on the survey protocol, questionnaire administration, sample collection and transport of biological samples. The international agency, FHI, provided technical assistance for implementing IBBA. Appropriate ethical clearances were obtained prior to the surveys. Further details of IBBA methodology are available in the papers published earlier [18,19,23,24].

#### c. STI clinical quality monitoring assessments

A major component of Avahan HIV prevention services was to ensure provision of high quality and standardized STI services through implementation partners [25]. An FHI led STI capacity building team developed a clinical quality monitoring tool. The quality monitoring assessments were implemented in a participatory manner at Avahan clinics at quarterly intervals by an external team in Maharashtra. The detailed methodology for these assessments has been described elsewhere [21]. Total quality scores were calculated and correlation matrix was used to examine significant change in scores between years using STATA 11® (Stata Corporation, College Station, TX).

### Operational definitions and assumptions

#### 1. Was coverage of Avahan adequate?

Coverage was defined on the basis of availability, utilization and coverage of HIV prevention services for FSWs [16,26]. The adequacy of coverage was defined based on the Avahan target for saturated coverage, set at 80% of the estimated denominator of the FSW population. This estimate of FSWs determined in March 2009 in the intervention districts was used as the intended denominator to be covered for the entire period examined in this assessment [16,22].

The Avahan target for outreach contacts was a minimum of one contact per month, whereas the target for clinic visits was once per quarter (about 33% of the total denominator per month) for STI consultations. The CMIS data were validated (‘evaluated coverage’) by comparing with corresponding IBBA data.

Intensity was defined as the frequency of exposure to Avahan program services measured based on the indicators listed in the framework [17] Staffing to achieve program intensity was measured using the indicators as mentioned in the framework [17] (the program target ratio was 1:50) [27]. Free condoms distributed to FSWs by the program were tracked yearly from the Avahan CMIS and annual condom sales data from the condom social marketing program [20] in Maharashtra. Data on the reported sources of obtaining condom last time from IBBA were used to validate trends from the CMIS.

#### 2. Has there been an increase in reported condom use in high risk groups?

Self-reported condom use from two rounds of IBBAs was used to assess changes in condom use patterns with commercial and non-commercial partners of FSWs. Consistent condom use was defined as condom use every time and no reported unprotected sex acts defined as condom use every time with both occasional and regular clients.

#### 3. Has there been a reduction in STIs and new HIV infections?

Changes in STI prevalence were measured based on tests done on blood and urine samples collected from FSWs during IBBAs. Biological tests included syphilis serology using Rapid Plasma Reagin (RPR) and confirmatory *Treponema pallidum* Hemagglutination Assay (TPHA), and nucleic acid amplification test (Gen-Probe APTIMA COMBO 2) for urine samples for chlamydia and gonorrhoea prevalence [18]. Any positive RPR confirmed with TPHA was defined as reactive syphilis or lifetime syphilis; whereas RPR yielding titres ≥1:8 or more were defined as active or high-titre syphilis.

HIV prevalence was assessed from HIV sero-prevalence data from two rounds of IBBAs. HIV positivity was determined using a two-test algorithm using enzyme immunoassay (J. Mitra EIA kit) [18]. As a proxy for new HIV infections, HIV prevalence among newer FSWs, those who entered the sex work profession in the last one year, and among young FSWs between the ages of 18 and 20 years (FSWs younger than 18 years old were excluded from the survey), were examined. For district level analysis in Mumbai and Thane, young age was considered to be less than 25 years for getting statistical power in the analysis.

#### 4. Association of Avahan exposure to increasing condom use and declining STIs

A composite indicator of exposure, having received any of these above 3 services was used for the analysis [17]. Pooled IBBA data from the two rounds were used to examine the association between exposure to Avahan interventions and condom use and STIs. Exposure to any intervention (either Avahan or non-Avahan) was also defined similarly as above.

##### Data management and statistical methods

District–level double-data entry was performed using CSPro Software (U.S. Census Bureau, Washington DC) during both rounds of IBBA. SPSS 15.0 statistical software was used for data analysis. District-level data of each round were merged to generate a state-level data set for Round 1 and Round 2. For some analyses, these merged data from Round 1 and 2 were aggregated to obtain pooled data. Appropriate weights were calculated and used for analysis of the district and state level datasets for both Round 1 and Round 2 [22]. Bi-variate and multivariate analyses were done using the complex samples module in SPSS 15. Wald Chi-square test was used to assess significant changes in profile characteristics among FSWs between the two rounds of IBBA. Multivariate logistic regression was used to assess significant changes in a) exposure measures, b) condom use outcomes with different partner types, and c) prevalence of HIV and other STIs, between the two IBBA rounds as well as for studying the association between exposure to Avahan services and having any STI (NG, CT or high-titre syphilis) and consistent condom use with commercial and non-commercial partners. Special analysis of Mumbai and Thane districts combined was compared with the other IBBA districts combined to better understand changes in HIV prevalence. Profile variables found to be significantly different in bivariate analysis between two IBBA rounds (Table 2) were adjusted in logistic regression models to generate adjusted odds ratios (AORs) and their significance was tested using the Wald-test. Z-test was applied to test significant changes in district level analysis. Associations were considered significant for p-values lower than 0.05.

**Table 2:** Socio-demographic and sex work characteristics of FSWs in Maharashtra in Rounds 1 and 2 of IBBAs

Characteristic	Categories	Round 1 % (n=2525)	Round 2 % (n=2525)	P value (Wald-Pearson test)
Current age (years)	< 25	20.9	23.0	0.602
	25-29	28.4	26.2	
	30-34	22.1	20.8	
	35-39	12.9	14.9	
	40+	15.8	15.1	
	
	Mean	**30.6**	**29.9**	

Literacy	Illiterate	75.6	74.3	0.572

Marital status	Ever married	79.2	81.4	0.358
	Never married	20.8	18.6	

Additional Income	Yes	6.6	7.6	0.450

Residency	Local dweller	98.1	92.6	0.000

Age at first sex (years)	< 15	48.8	46.4	0.365
	15+	51.2	53.6	
	
	Mean	**16.85**	**17.51**	

Age started sex work (years)	<20	31.9	27.8	0.011
	20-24	34.6	34.9	
	25-29	21.2	19.2	
	30+	12.2	18.2	
	
	Mean	**22.65**	**23.77**	

Duration sex work (years)	0-1	18.0	18.8	0.438
	2-4	17.8	21.0	
	4-9	32.6	31.5	
	10+	31.6	28.8	
	
	Mean	**7.41**	**6.22**	

Usual place of solicitation	Non street based	68.9	69.3	0.909
	Street based	31.1	30.7	

Usual place of entertaining clients	Home	26.7	33.0	0.043
	Brothel/lodge/dabha*	72.5	66.6	
	Public places	0.9	0.3	

Commercial clients per week	0-4	18.7	14.8	0.065
	5-9	33.7	31.8	
	10+	47.6	53.4	
	
	Mean	**11.89**	**16.73**	

Has regular partner	Yes	31.4	49.5	0.000

Source of obtaining condom	Peer educator/out reach worker/NGO	49.9	62.3	0.000
	Other source	50.1	37.7	

Frequency of peer contacts in a month	None	9.1	5.8	0.001
	1-2 times	17.2	27.4	
	3-4 times	73.7	66.8	
	
	Mean	**1.6**	**1.6**	

*Dabha – road-side restaurants on highways are commonly referred to as dabha and often serve as halt points for trucks.

## Results

A total of 5,100 FSWs (2,525 in Round 1 and 2,575 in Round 2) were sampled in the two rounds of IBBAs [25] However, for this analysis Parbhani FSWs were excluded as the sampling methodologies adopted in Round 1 and Round 2 were different. In Round 1 respondent driven sampling method was applied whereas in Round 2 it was take-all approach. Table 1 gives the number of sampled FSWs per district. Table 2 provides the profile characteristics of FSWs in Round 1 and Round 2. Overall about 5.8% of FSWs in Round 2 reported that they also participated in Round 1 IBBA.

### Is coverage of Avahan adequate?

#### Scale up of Avahan program

**Figure 2:**
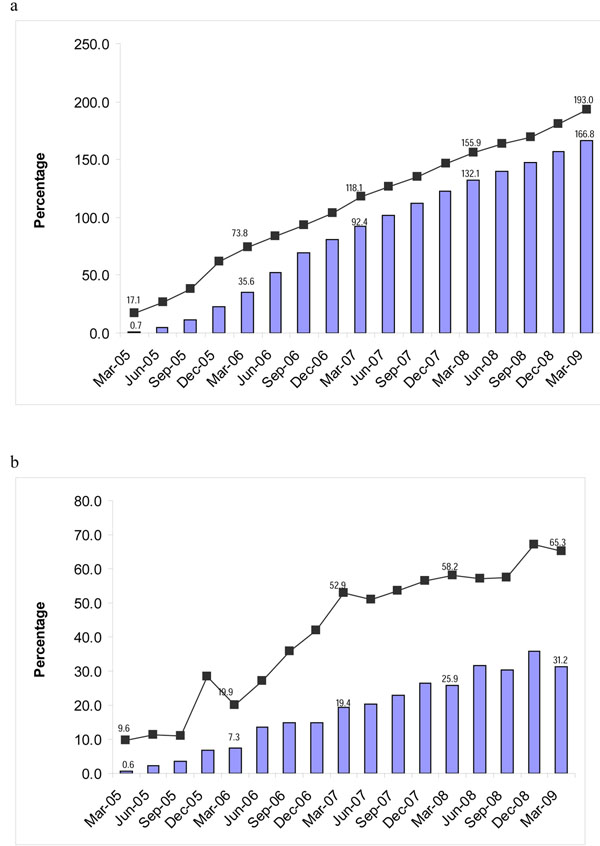
a) Proportion of FSW ever contacted and ever visited clinic in Maharashtra (Avahan CMIS 2005-2009). b) Proportion of FSWs contacted and visiting clinic in a month in Maharashtra (Avahan CMIS: 2005 - 2009)

The Avahan program for FSWs in Maharashtra began in 2004 in three districts and scaled up to 16 districts by 2008 through 32 NGOs working under the three state lead implementing partners. The estimated number of FSWs planned to be reached by March 2009 was about 47,650. By the first year of program implementation, 68% of the estimated FSW denominator was contacted at least once and 11% had visited STI clinics at least once. This increased to over 193% (outreach contacts) and 167% (STI clinic visits) of the estimated FSW denominator by March 2009 (Figure 2).

The proportion of FSWs contacted monthly by peer educators increased from 28% in December 2005 to 66% in March 2009 (Figure 2) while monthly STI clinic visits increased from 1% to 34% in the same period (Figure 2).

Analysis of IBBA data showed the proportion of FSWs who were ever contacted by Avahan peer educators increased from 26% in Round 1 to 52% in Round 2 (AOR: 2.34; p<0.001) and, similarly, visits to Avahan STI clinics increased from 28% to 45% (AOR: 1.6; p<0.001). The proportion of FSWs who were contacted by Avahan peer educators in the last month also increased significantly from 30.1% in Round 1 to 46.6% in Round 2 (AOR: 1.64, p<0.001).

#### Intensity of coverage

**Figure 3:**
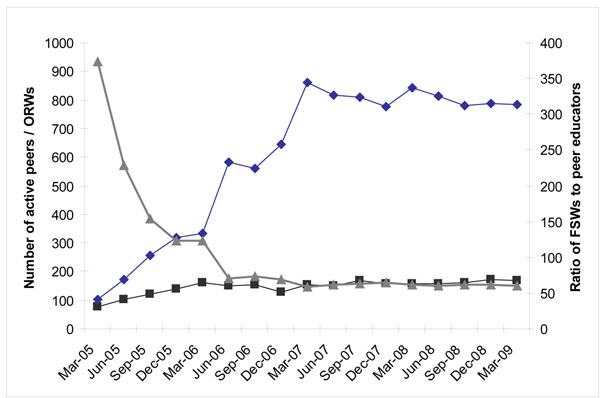
Number of active peers educators / outreach workers and ratio of FSWs to peers educators Maharashtra (Avahan CMIS 2005-2009)

With geographical expansion of the Avahan program, the numbers of peer educators increased nearly eight-fold and the number of outreach workers more than doubled between 2005 and 2009 ( Figure 3). The ratio of peer educator to FSWs reached 1:58 by March 2007 and has remained stable.

Annual condom distribution by the Avahan program in Maharashtra increased steadily from about 2.6 million condoms in 2005 to about 19.3 million condoms in 2008. In the same period, program-supported condoms sales in Avahan districts increased from over 10.6 million in 2005 to about 31 million in 2008. The number of condoms distributed or sold by the program per FSW per month increased from 19 in 2005, to 43 in 2007 and to 54 in 2008. The number of commercial sex acts per FSW per month was 44 in both 2006 and 2008 based on IBBA, indicating that, based on program data, sufficient condoms were being distributed by the program to cover the estimated commercial sex acts by 2008. The proportion of FSWs reporting during IBBA to having received condoms from Avahan increased from 50% in Round 1 to 62% in Round 2 (p<0.001) (Table 2).

**Figure 4:**
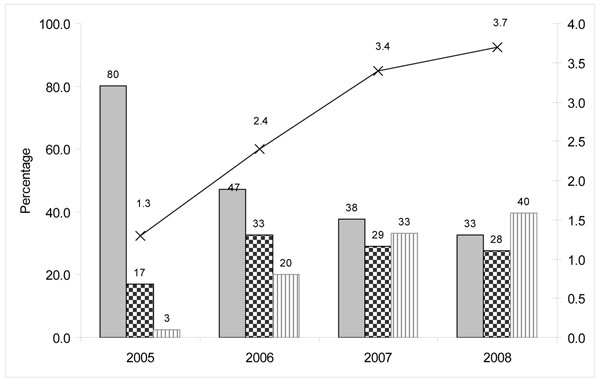
Frequency of visits by FSWs in Avahan program clinics in Maharashtra (Individual STI clinic data: 2005-2008)

IBBA data on the frequency of contacts revealed that peer educators reached FSWs at least once a month in both rounds. The percentage of FSWs not contacted in the last month declined from 9.1% in Round 1 to 5.8% in Round 2 (Table 2). MIS clinic data indicated that the proportion of FWS making four or more visits to Avahan STI clinics increased from 3% in 2005 to 40% in 2008, while the proportion of FSWs making only one visit declined from 80% in 2005 to 33% in 2008. The average number of clinic visits per FSW increased from 1.3 in 2005 to 3.7 in 2008 (Figure 4).

#### Quality of service

Quality assessments covered an average of 12 Avahan STI clinics per year. The total number of Avahan clinics increased from 37 to 93 static and preferred provider clinics between 2005 and 2008. These revealed a consistent improvement in quality scores from 2005 to 2008 in all components of the clinic service such as clinic management (1.6 to 3.8; p=0.07), clinic performance (0.9 to 3.2; p=0.05) and clinic operation (2.0 to 3.9; p=0.12) with total scores increasing from 1.5 to 3.6 out of maximum of 5.0 (p=0.07).

### Has there been a change in condom use among high risk groups?

The proportion of FSWs who reported having no risky sex acts (without condoms) increased from 72% in Round 1 to 95% in Round 2 (Table 3). The reported consistent condom use with both occasional and regular clients increased significantly in IBBA Round 2 compared to Round 1 (Table 3), after adjusting for confounding in logistic regression analysis. In contrast, consistent condom use with regular partners showed a significant decline to 13.7% in Round 2 as compared to 21.2% in Round 1 (Table 3).

**Table 3:** Univariate and multivariate analysis of condom-related outcomes and biological indicators in round 1 and 2 of IBBA in Maharashtra

*Condom-related outcomes and biological indicators*	Round 1 (n=2525) %	Round 2 (n=2525) %	Crude OR (95% CI)	Adjusted OR (95% CI)	P value for adjusted OR (Wald test)
***No reported unprotected sex with commercial clients***	76.2	94.6	5.50** (3.61-8.40)	5.10 (3.38-7.73)	0.000
***Condom use last sex with occasional clients***	96.8	99.7	9.41** (4.13 – 21.44)	7.78 (3.17 – 19.13)	0.000
***“Every time” condom use with occasional clients***	83.3	96.5	5.54** (3.15 – 9.74)	4.99 (2.97 – 8.38)	0.000
***Condom use last sex act regular clients***	81.1	99.1	25.27** (9.36 – 68.21)	41.82 (17.28 – 101.26)	0.000
***“Every time” condom use with regular clients***	78.3	94.5	4.80** (3.01 – 7.66)	4.53 (2.97 – 6.90)	0.000
***Consistent Condom use with regular partner* ^+^**	21.2	13.7	0.58** (0.41 – 0.84)	0.63 (0.44 – 0.89)	0.010
***HIV***	25.8	27.5	1.08 (0.85 – 1.38)	1.29 (1.00 – 1.65)	0.044
***Syphilis***	15.8	10.8	0.64** (0.480 – 0.866)	0.54 (0.41 – 0.72)	0.001
***Syphilis high titre***	4.2	3.4	0.81 (0.49 – 1.33)	0.56 (0.35 – 0.89)	0.016
***Chlamydia infection***	8.0	6.2	0.76 (0.53 – 1.10)	0.65 (0.47 – 0.90)	0.010
***Gonorrhoea infection***	7.4	3.9	0.51** (0.34 – 0.76)	0.60 (0.47 – 0.90)	0.026
***Chlamydia and/or Gonorrhoea infection***	12.7	9.3	0.70** (0.51 – 0.95)	0.67 (0.50 – 0.91)	0.010

**<0.01 *<0.05. Multivariate models were adjusted for the following variables: District, Literacy, Marital Status, additional sources of income, Locality status, Duration in sex work, Usual place of solicitation, Usual place of entertaining clients, No. of Clients per week and Proportion of FSWs with regular partners.

### Have there been changes in STIs and new HIV infections among HRGs?

Between Round 1 and Round 2 of the IBBAs, the prevalence of reactive syphilis serology declined from 15.8% to 10.8% and high-titre syphilis reduced from 4.4% to 3.4% (Table 3). Prevalence of chlamydia and gonorrhoea also significantly declined between the two rounds (Table 3).

Overall HIV prevalence among FSWs increased significantly from 25.8% to 27.5% between Round 1 and Round 2 (Table 3). By district, significant increases in HIV prevalence were observed in Mumbai and Thane districts while HIV prevalence declined in other districts such as Kolhapur and Pune among brothel- and non-brothel-based FSWs, and in Yevatmal after controlling for confounding factors (Table 4).

**Table 4:** District-level HIV prevalence in Rounds 1 and 2 of IBBAs in Maharashtra

District/group/combination of district	Round 1 % (N)	Round 2 % (N)	AOR (p-value)
***Kolhapur***	**33.0 (115)**	**27.4 (190)**	**1.1 (0.732)**
**Mumbai brothel**	**28.1 (407)**	**34.9 (395)**	**1.4 (0.08)**
**Mumbai street**	**19.2 (394)**	**32.3 (385)**	**3.1 (0.002)**
**Pune brothel**	**38.7 (404)**	**20.3 (403)**	**0.5 (0.001)**
**Pune non-brothel**	**37.0 (257)**	**22.0 (266)**	**0.5 (0.024)**
**Thane brothel**	**18.6 (401)**	**33.1 (384)**	**2.3 (0.001)**
**Thane street**	**7.0 (394)**	**11.5 (395)**	**1.7 (0.19)**
**Yavatmal**	**37.3 (153)**	**26.8 (157)**	**0.6 (0.191)**
**All district combined**	**25.8 (2525)**	**27.5 (2575)**	**1.3 (0.044)**
**Mumbai and Thane combined**	**23.6 (1596)**	**30.6 (1203)**	**1.8 (0.000)**
**All district except Mumbai Thane combined**	**36.9 (929)**	**21.1 (1372)**	**0.05 (0.000)**

Logistic regression analysis of combined Mumbai and Thane districts compared with other Maharashtra districts combined revealed that HIV prevalence in Mumbai-Thane increased significantly whereas a decline was seen in the “other-districts” domain, after controlling for confounding factors (Table 4). In Mumbai HIV prevalence increased among FSWs less than 25 years, from 15.2% in Round 1 to 38.9% in Round 2 (p=0.001), stable among new FSWs, <=1year, (19.1% in Round 1 and 19.2% in Round 2 but increased among FSWs in sex work for longer duration (Table 5). In Thane district, HIV prevalence increased among younger FSWs as well as new FSWs (Table 5). Coverage by any interventions in Mumbai increased significantly among sub-groups of FSWs by age and duration in sex work between IBBA rounds (Table 6). Whereas in Thane district no difference in coverage was observed by age groups but coverage increased significantly among new entrants and declined among FSWs in the profession for longer duration (Table 6).

**Table 5:** HIV prevalence (%) among FSWs in Mumbai and Thane Districts by age and duration in sex work in IBBA Round 1 and Round 2, Maharashtra

Indicators	Mumbai			Thane		
	
	R1 N=801	R2 N=780	p-value	R1 N=	R2 N=	p-value
**Current age**						

< 25 years	15.2	38.9	0.000	6.8	19.7	0.000
25-29 years	25.3	29.1	0.22	10.5	21.6	0.001
30-34 years	30.6	33.2	0.37	20.3	26.3	0.135
35-39 years	20.0	34.7	0.004	27.9	22.5	0.313
40 years and above	23.6	34.3	0.039	18.9	23.6	0.29

**Duration in sex work**						

< = 1 years	19.1	19.2	0.43	7.5	16.3	0.013
2 – 4 years	16.6	40.9	0.000	12.7	12.2	0.44
5 – 9 years	23.9	33.2	0.015	15.4	27.3	0.000
10 years and above	29.2	36.5	0.024	16.1	37.6	0.000

**Table 6:** Coverage of FSWs (%) by any interventions in Mumbai and Thane districts by age and duration in sex work in IBBA Round 1 and Round 2, Maharashtra

Indicators	Mumbai			Thane		
	**R1** **N=801**	**R2** **N=780**	**p-value**	**R1**	**R2**	**p-value**

**Current age**						

< 25 years	32.1	58.3	0.000	53.9	57.7	0.292
25-29 years	36.0	70.9	0.000	58.5	63.3	0.209
30-34 years	41.9	65.7	0.000	60.9	61.7	0.453
35-39 years	35.1	77.5	0.000	76.9	65.6	0.109
40 years and above	39.4	72.0	0.000	72.4	62.3	0.133

**Duration in sex work**						

< = 1 years	19.4	39.3	0.000	32.3	64.0	0.000
2 – 4 years	31.3	75.1	0.000	56.4	65.8	0.032
5 – 9 years	35.5	71.9	0.000	72.1	54.1	0.000
10 years and above	54.3	76.6	0.000	82.4	63.5	0.000

Stratification of HIV-positive FSWs in all districts, by age and duration of sex work revealed a marginal decline in the percentage of HIV-positive sex workers who were new in sex work (less than one year of duration) but was not found to be significant (Round 1: 17.9%; Round 2: 16.6, p=0.78). However, the proportion of young sex workers (aged between 18-20 years) who were HIV-positive increased significantly from 8.8% in Round 1 to 23.2% in Round 2 (p=0.04).

### Is Avahan program exposure associated with changes in condom use and STI prevalence?

The proportion of FSWs who had no unprotected sex was significantly higher among FSWs exposed to Avahan (72%) compared to unexposed FSWs (58%). Similarly, consistent condom use with both occasional and regular clients was significantly higher among FSWs exposed to Avahan (Table 7).

**Table 7:** Univariate and multivariate analysis of condom-related outcomes and biological indicators among FSWs exposed and unexposed to Avahan intervention in Maharashtra

*Indicators*	Unexposed	Exposed	Crude OR (95% CI)	Adjusted OR (95% CI)	P value (Wald test) Adjusted OR
***No reported unprotected sex with commercial clients***	57.9 (n=2509)	72.0 (2591)	1.870** (1.404 – 2.492)	1.596 (1.185-2.149)	0.002
***Condom use last sex act with occasional clients***	97.8 (n=2406)	98.9 (n=2501)	2.07 ** (1.24-3.47)	2.64 (1.40-4.98)	0.003
***Consistent condom use with occasional clients***	90.6 (n=2406)	94.3 (n=2501)	1.74 ** (1.08-2.77)	1.55 (1.02-2.34)	0.039
***Condom use last sex act with regular clients***	97.4 (n=1946)	98.3 (n=2233)	1.53 (0.89-2.63)	1.46 (0.71-3.03)	0.305
***Consistent Condom use with regular clients***	86.0 (n=1946)	92.5 (n=2233)	2.02** (1.34-3.05)	1.95 (1.33-2.85)	0.001
***Consistent Condom use with regular partner* ^+^**	16.1 (n=1013)	17.0 (n=1185)	1.07 (0.74-1.53)	1.23 (0.83-1.84)	0.300
***Gonorrhoea infection***	6.7 (n=2525)	4.2 (n=2575)	0.60** (0.41-0.88)	0.76 (0.51-1.17)	0.218
***Chlamydia infection***	7.4 (n=2525)	6.7 (n=2575)	0.89 (0.62-1.28)	0.90 (0.64-1.27)	0.560
***Syphilis***	14.6 (n=2525)	11.4 (n=2575)	0.75** (0.57-0.98)	0.58 (0.44-0.77)	0.000
***High-titre Syphilis***	3.9 (n=2525)	3.6 (n=2575)	0.93 (0.57-1.50)	0.58 (0.33-1.03)	0.063
***Gonorrhoea/chlamydia***	11.9 (n=2525)	9.8 (n=2575)	0.79 (0.590-1.083)	0.88 (0.66-1.19)	0.426
***Any STI (gonorrhoea or chlamydia or high-titre syphilis)***	15.4 (n=2525)	12.7 (n=2575)	0.80 (0.61-1.04)	0.77 (0.58-1.02)	0.067

**<0.01 *<0.05. Multivariate models were adjusted for the following variables: District, Literacy, Marital Status, additional sources of income, Locality status, Duration in sex work, Usual place of solicitation, Usual place of entertaining clients, No. of Clients per week and Proportion of FSWs with regular partners

^+ :^ Variable on FSWs reporting having regular partner was not controlled in the model

The prevalence of any STI (high-titre syphilis, NG or CT) among FSWs exposed to Avahan services was 12.7% compared with 15.4% among unexposed FSWs (AOR: 0.7; p=0.67). The prevalence of reactive syphilis among unexposed FSWs was 14.6% compared with 11.4% among FSWs exposed to Avahan (AOR: 0.58; p<0.001) (Table 7).

## Discussion

The current paper presents one of the first efforts at assessment of a large scale prevention program for female sex workers in the state of Maharashtra. The analysis in the paper provides evidence that the Avahan program in Maharashtra targeting female sex workers was rapidly scaled up to reach more than two thirds of the FSWs, and that this coverage was associated with increased condom use and reduced STIs among those who were exposed to the intervention.

In India, other than the NACO led mid-term assessment, there are few large scale assessment studies available on the outcomes and impacts of HIV prevention interventions among FSWs. Assessment of the Avahan program in other Indian states provided similar results as presented here [17,19]. Other than these, an assessment of the Sonagachi project among FSWs in Calcutta [28] compared the outcomes in project areas and NACO intervention areas and found no difference. In both areas, condom use increased and prevalence of STIs declined. Assessments in geographic areas with multiple program players make it difficult to tease out the effectiveness, particularly among mobile populations who are free to receive services from any program and also have potential for diffusing the effect of interventions. Assessment of sex worker interventions, in South-east Asian and African countries [29] have shown improvements in condom use and declines in STIs using similar strategies, however these were smaller scale studies.

The Avahan program for FSWs in Maharashtra was scaled up steadily and achieved coverage of 66% of the estimated number of FSWs within four years of implementation. As the proportion of ‘ever contacted’ and ‘ever visited clinics’ counted FSWs contacted or visited clinic at least once, it went beyond 100%. This suggests a high degree of mobility and turnover of FSWs in the coverage districts.

The monitoring data showed a progressively wider geographical area was covered. The program improved required infrastructure to achieve increased contacts with FSWs through outreach and clinical services for STIs, which was validated using IBBA data. The difference in coverage estimates from monitoring data and IBBA are likely due to two main reasons. By the time IBBA Round 2 was conducted, a number of districts were in the process of partially or fully being transitioned to NACO and therefore would have affected the measurement of coverage (contacts in the last month). Additionally, the high levels of mobility and turnover of FSWs in Maharashtra districts have been reported previously [31] and therefore would also affect the measurement of coverage indicators. These findings are also consistent with the MIS data, discussed above, that the proportion of ever contacts was well over 100%.

The minimum requirements for condoms were achieved by Avahan through free distribution or social marketing to cover the estimated commercial sex acts by 2008. Other studies have found similar results supporting the claim that the condom supply was increased by Avahan [20]. The increase in peer educators as the source of condom by FSWs reported during the IBBA validated the CMIS condom distribution data. Other than the Avahan program, free condom distribution and social marketing was taken up as an important component of the National AIDS Control Program in all states indicating that more than sufficient condoms were available to HRGs [32,33].

Significant improvements in condom use with commercial partners of FSWs were seen between IBBA Rounds 1 and 2. High levels of consistent condom use were reported with both occasional and regular clients similar to the patterns seen by other studies elsewhere in India [34]. Increasing trends in condom use with paying clients have been noted in other Behavioural Surveillance Studies in Maharashtra [35] and other BSS studies in India [36]. The main area of concern is the low use of condoms with regular partners, which has not improved over time. The low rates of reported condom use with regular partners have been reported previously, and are due to factors such as trust, intimacy and low risk perceptions, avoidance of suspicion of being a sex worker, power relationships with spouses and partner refusal and violence [33,34,36,37]. Additional programmatic focus addressing these factors, innovative communication strategies and facilitating a supportive environment can help to further improve condom use with regular partners of FSWs.

A significant decline in STIs, including reactive syphilis, gonorrhoea and chlamydia, is an important finding. The decline in the prevalence of STIs, particularly syphilis, was associated with exposure to Avahan. Clinic MIS data are consistent with this and indicate a decline in symptomatic STIs over time with improvements in regular check-ups screening and management of STIs [39]. Monthly screening and treatment of symptomatic and asymptomatic sex workers as practiced in Avahan [21], has been shown to be effective in reducing STIs among sex workers previously [39]. A similar reduction in prevalence of one or more of STIs has been observed in the southern state of Karnataka and was associated with Avahan program exposure [34,40,41].

Although HIV prevalence among FSWs in overall Maharashtra increased significantly over time, a significant decline was observed in three of the districts. Studies in Pune district in Maharashtra have indicated a nearly similar trend in HIV prevalence among FSWs. A study among FSWs attending STI clinics in Pune reported stable HIV prevalence (between 46% to 50%) among FSWs over a 10-year period [42] while a prospective cohort study also in Pune found declining incidence of FSWs and males with STIs attending STI clinics over a 10-year period [43].

A closer examination of IBBA data indicated that the increase was mainly due to two districts: Mumbai and Thane. Though the HIV sentinel surveillance indicates that the mean prevalence of HIV among FSWs has declined from 54.3% in 2003 to 17.9% in 2007, in Maharashtra [44,45], HIV prevalence in FSW sites in Mumbai and Thane remained high compared to other districts (>30%) consistent with results in this study. In this study it was also found that overall in the state HIV prevalence among young sex workers (between 18 to 20 years) increased. In Mumbai district prevalence increased among new entrants into sex work (<=1 year) and in Thane district among younger FSWs (<25 years) and new into sex work though the coverage of interventions also increased between IBBA rounds. This suggests that the interventions are not reaching the younger and vulnerable FSWs soon enough.

High HIV prevalence (nearly 25%) was reported in earlier studies [46] among women and girls who were trafficked. Mumbai has been reported to have the largest centre for sex trafficking in India [46] and with little more than half of those being under-age or aged below 18 years [47]. Other reasons for increased HIV prevalence in Mumbai and Thane are likely due to the context of these districts. Both are large metropolitan areas with a population between 8 and 13 million, and are a major centre of economic activities with large numbers of mobile migrant workers and FSWs. Mumbai is well established to be a HIV epicentre in India, a major destination for FSWs from other parts of India with high concentration of HIV-infected high-risk populations, [2,11,13,31] and high mobility of high-risk groups and their clients between Mumbai and Thane is also well established, given that they are neighbouring cities.

Information from the programs in Mumbai and Thane suggests that the turnover rate of FSWs ranges from 20% to 25% annually [personal communication from V. Ranebennur]. A study on the mobility of FSWs in Maharashtra found that more than 50% of FSWs had reported moving at least twice in a two-year period and mobility across state was highest among FSWs from Mumbai and Thane districts [31]. Another possible reason for increased prevalence could be that an increasing number of HIV-positive FSWs are obtaining anti-retroviral treatment (ART) locally. While data specifically on FSWs accessing the free government ART services are not available, the increasing trend of adult ART registrations during this period may have also contributed to the increased HIV prevalence [33,44].

Assessment of interventions providing similar package of services in Cambodia have shown decline in prevalence of HIV among FSWs [48,49]. However examples of continued high rates STIs and/or HIV among FSWs despite increased condom use have been also reported in Thailand and Cambodia [48-50]. This is likely due to factors such as high mobility and turnover of FSWs, factors such as violence and forced sex [50,51] and most importantly, the low level of condom use with non-commercial partners who are not effectively addressed by prevention programs and contribute to further HIV and STI transmission.

Further data on estimates of HIV incidence among FSW in these districts can provide additional insights for these trends. Additional studies are also required to better understand the vulnerabilities of younger FSWs and new entrants to sex work, and strategies for reaching them early, to inform programmatic interventions.

The association of Avahan program exposure with condom use by FSWs with clients is indicative of a positive influence of Avahan intervention on behaviour change among FSWs. Assessment studies of peer-mediated interventions in other countries [29,52], the Sonagachi program in India [28] as well as those implemented by Avahan in other states have shown that peer-mediated strategies are effective in increasing condom use among FSWs [34].

This assessment also found that within four years, FSWs exposed to Avahan were less likely to have active syphilis while the likelihood of not having any STI (high-titre syphilis, gonorrhoea or chlamydia) was significant at the 0.1 level. Studies in other states have reported evidence for a stronger association between Avahan exposure and reductions in STI prevalence [19,34]. The other evidence in India similar to this study comes from the Sonagachi project, which reported a decline in active syphilis from 4.8% to 1.2%, nearly 10 years after baseline [28].

While the scope of the present assessment was not to assess the impact of Avahan program at the general population level in Maharashtra, some initial observations for assessing impact can be made. HIV prevalence among pregnant women attending antenatal clinics (ANCs) in sentinel surveillance sites in Maharashtra has been declining steadily from about 1% in 2004 to 0.64% in 2008 [44,45] similar to changes seen in other parts of India and at the national level [45]. Similarly, in districts where Avahan interventions were implemented, the prevalence among ANC attendees declined from about 1.2% in 2003 to 0.98% in 2007 [43] and further down to 0.75% in 2008 [33,53]. Data from pregnant women attending prevention of parent-to-child transmission of HIV (PPTCT) clinics also showed a declining trend [54]. HIV prevalence among PPTCT clinics has declined from 1.54% in 2003 to 0.5% in 2008 [11,54]. HIV prevalence among individuals at voluntary testing and counselling sites in Maharashtra has also declined from 1.1% in 2001 to 0.54% in 2007 [11].

Assessment of ANC data from other Avahan states, such as Karnataka [55] have showed a significant decline in HIV prevalence among ANC attendees in Avahan intervention districts compared with non-intervention districts. However, unlike Karnataka, Maharashtra had multiple implementation players in any given district and the state, thus assessment is more complex and any impact observed would point to the combined impact of interventions.

There are several limitations to this assessment. The round 1 of the survey was carried out when program just initiated thus it was not a true baseline for Avahan program. Self-reported condom use may be biased due to socially desirable responses from survey participants. In the current surveys, the self-reported responses to different condom use questions were found to be internally consistent and were in the expected relative frequency by partner type but, without additional information, we are unable to assess the degree of social desirability bias. Other studies that have reported increases in consistent condom use among clients of FSWs from hotspots in Maharashtra from 84% in 2006 to 96% in 2008, which provides some confidence in the validity of our findings [56].

Another limitation is that Avahan was designed to increase the coverage of FSWs. In the case of Maharashtra especially, this meant that Avahan was supporting programs in districts where other interventions existed. The IBBA used the entire district as the sampling frame while the MIS data provided information on FSWs only in Avahan catchment areas. Further, the assessment design did not provide any control groups, in consideration of ethical issues of withholding critical STI services, high mobility and diffusion, plans for rapid scale-up and transferring the program to government. Therefore the outcomes of this assessment cannot be attributed to Avahan interventions directly. Moreover, lack of comparable district-level data on outcomes and intervention exposure among FSWs in other non-Avahan districts makes it difficult to carry out any further analysis comparing Avahan and non-Avahan areas.

The current assessment was therefore based on recent approaches recommended and appropriate for large-scale public health programs, with complex multi-player situations [57-60]. The strength of this current analysis is having evidence along the framework of program logic and having independent and multiple rounds of cross-sectional survey data from the same geographic areas that indicate congruency of trends, adding to the evidence for program effectiveness.

The overall increase in the prevalence of HIV in the state of Maharashtra is alarming and further analysis at district level has revealed that it is mainly due to the high significant increase in HIV prevalence observed in the district of Mumbai and Thane. Further in-depth analysis specifically for the two groups viz. brothel-based FSWs in Thane and street based FSWs in Mumbai is suggested taking into account the other available data sources for better understanding and to answer the sudden sharp increase in these groups. Also, tailored and sustained programmatic efforts are needed to address the disparity in the prevalence of HIV and STI in the districts of Maharashtra.

## Conclusion

In conclusion, Avahan successfully implemented a large-scale HIV prevention program among FSWs in Maharashtra amidst longstanding HIV prevention programs, multiple intervention players, high levels of population turnover and achieved high coverage within four years of implementation. The increased coverage and intensity of the program was associated with improvements in condom use and declines in STIs. HIV prevalence among FSWs decreased in three districts but increased in Mumbai and Thane districts. Declining prevalence among general population groups has been seen in the state but given the complexity of the environment and multiple players, the impact of program cannot be isolated and should be seen as the combined impact of multiple interventions.

## Competing interests

The authors declare that they have no competing interests.

## Author’s contributions

RSP was the principal investigator of the study. He provided continuous guidance from the development of the concept till the finalization of the manuscript. MM was the key person who coordinated the study and contributed in concept development and analysis; SD was involved in the data collection and provided insights from the field work; SK was accountable for the biological test results and contributed in the concept development; AG was responsible for coordinating the study, concept development, analysis, writing and finalization of the manuscript; PG was the involved in concept development and analysis; SR contributed to the drafting and formulation of framework; RA guided in planning and execution of study, development of concept and framework design; BG contributed in finalization of concept, framework design and finalization of manuscript.
